# Identification of a unique Ca^2+^-binding site in rat acid-sensing ion channel 3

**DOI:** 10.1038/s41467-018-04424-0

**Published:** 2018-05-25

**Authors:** Zhicheng Zuo, Rachel N. Smith, Zhenglan Chen, Amruta S. Agharkar, Heather D. Snell, Renqi Huang, Jin Liu, Eric B. Gonzales

**Affiliations:** 1 Department of Pharmaceutical Sciences, University of North Texas System College of Pharmacy, University of North Texas Health Science Center, Fort Worth, TX 76107 USA; 2 Department of Pharmacology & Neuroscience, Institute for Healthy Aging, University of North Texas Health Science Center, Fort Worth, TX 76107 USA; 3 Department of Medical Education, TCU and UNTHSC School of Medicine (Candidate for LCME accreditation), Fort Worth, TX 76129 USA

## Abstract

Acid-sensing ion channels (ASICs) evolved to sense changes in extracellular acidity with the divalent cation calcium (Ca^2+^) as an allosteric modulator and channel blocker. The channel-blocking activity is most apparent in ASIC3, as removing Ca^2+^ results in channel opening, with the site’s location remaining unresolved. Here we show that a ring of rat ASIC3 (rASIC3) glutamates (Glu435), located above the channel gate, modulates proton sensitivity and contributes to the formation of the elusive Ca^2+^ block site. Mutation of this residue to glycine, the equivalent residue in chicken ASIC1, diminished the rASIC3 Ca^2+^ block effect. Atomistic molecular dynamic simulations corroborate the involvement of this acidic residue in forming a high-affinity Ca^2+^ site atop the channel pore. Furthermore, the reported observations provide clarity for past controversies regarding ASIC channel gating. Our findings enhance understanding of ASIC gating mechanisms and provide structural and energetic insights into this unique calcium-binding site.

## Abstract (web summary)

Acid-sensing ion channels (ASICs) sense changes in extracellular acidity with Ca^2+^ as an allosteric modulator and channel blocker. Here authors use electrophysiology and molecular dynamics simulation to identify the residue in ASIC3 which modulates proton sensitivity and contributes to the Ca^2+^ block.

## Introduction

Acid-sensing ion channels (ASICs) are trimeric proton-activated sodium channels characterized by protein subunits comprised of intracellular amino-termini and carboxyl-termini, two transmembrane domains, and a large extracellular domain. These channels are part of the epithelial sodium channel (ENaC)/degenerin (DEG) family of ion channels^1^. In disease and human health, ASICs play a role in neurodegeneration following ischemia and inflammatory pain^2–6^. Four genes encode six ASIC subtypes (ASIC1a, 1b, 2a, 2b, 3, and 4), each exhibiting additional physiological functions^2,7–12^. In the central nervous system, ASIC1 acts as the primary proton receptor in the brain and has been implicated in stroke^13^, while the peripherally located ASIC3 primarily mediates the perception of pain^14,15^.

The gating behavior of many ASICs is intricately linked to the changes of both extracellular protons (H^+^) and calcium (Ca^2+^) concentrations. This interplay between cations has resulted in two distinct activation models: the relief of Ca^2+^ block and the allosteric gating mechanisms^16–20^. The Ca^2+^ block model^16,19^ proposes a high-affinity Ca^2+^-binding site sits at the extracellular side of the channel pore, and argues that proton-catalyzed Ca^2+^ release from this block site is sufficient to induce channel opening, accompanying no conformational change. This model was considered controversial at the time, but intriguing with the data shown. In particular, the model appears to work well for ASIC3 but cannot explain why disruption of the single Ca^2+^ block site does not constitutively open the channels^18^, suggesting an underdetermined gating manner independent of Ca^2+^ block. In the allosteric mechanism^16,17,20^, additional Ca^2+^/H^+^-binding sites are presumed to exist within multiple extracellular protein domains where conformational changes are caused by proton binding and Ca^2+^ unbinding, leading to channel opening. In support of the allosteric hypothesis, studies with ASIC1a have predicted a second Ca^2+^ binding/modulating site that is located near the wrist region and was shown to mediate Ca^2+^ regulation of proton gating^18^. Typically, the Ca^2+^ sensitivity is most unique in ASIC3 among the five ASIC subtypes, as simple removal of extracellular Ca^2+^ alone results in channel activation^19^, while the same removal does not result in ASIC1 opening in most cases^17,18^. Most likely, the Ca^2+^ block and allosteric effects contribute differentially to gating regulation among the ASIC family members. Noticeably, several conserved residues at the channel pore entrance have been implicated in Ca^2+^ binding^18,21–23^, however, little is known regarding the exact location of the putative site and its chemical principles for Ca^2+^ block action. Still, whether protons and Ca^2+^ compete for binding at one site or whether Ca^2+^ unbinding allosterically affects proton-induced activation remains to be further resolved.

Here, we investigate the gating mechanisms in ASICs by combining the electrophysiological and computational approaches, focusing on the chicken ASIC1 (cASIC1) and rat ASIC3 (rASIC3). We show that the unique Ca^2+^ sensitivity of rASIC3 is influenced by a ring of glutamate residues (rASIC3 Glu435) located above the channel gate. Mutation of this residue to glycine, the equivalent amino acid in cASIC1, diminishes the Ca^2+^ block effect in rASIC3. Correspondingly, the reverse substitution at the equivalent position in cASIC1, i.e., G429E, results in increased proton sensitivity and channel activation in the absence of Ca^2+^. Furthermore, our molecular dynamics simulations unveil the exact location of the Ca^2+^ block site and provide the structural and energetic characterization at this site. Our data provide strong evidence supporting the competition between H^+^ and Ca^2+^ at the gating site, and suggest that a pronounced local conformational change is necessary for channel opening accompanying proton-aided Ca^2+^ release.

## Results

### Relief of Ca^2+^ block activation involves ASIC3 (Glu435)

**Fig. 1:**
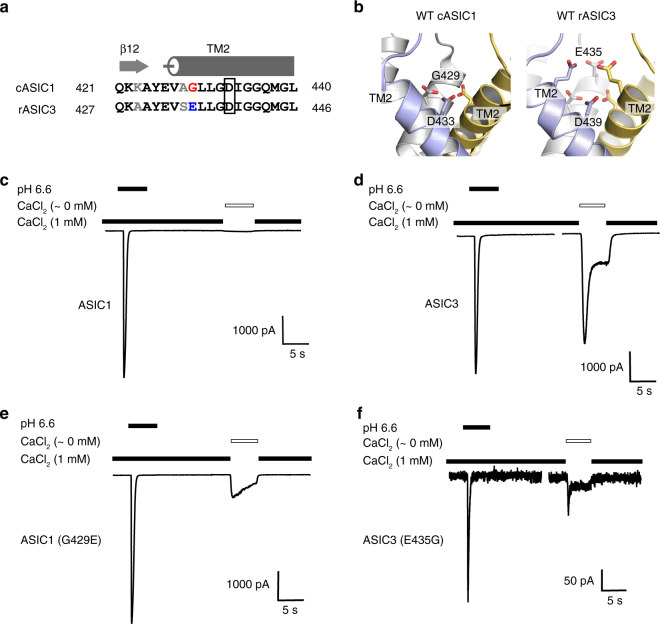
Mutation at the pore of ASIC3 mediates relief of Ca^2+^ block. **a** Sequence alignment of the β12 sheet and TM2 domains of cASIC1 and rASIC3. Residues that differ between these subtypes are shown in gray. The residues that contribute to the Ca^2+^ block site and studied in this report are highlight in red and blue. **b** cASIC1 (left) and modeled rASIC3 (right) protein gate regions are shown. rASIC3 was generated using the desensitized cASIC1 crystal structure (PDB code: 4NYK) as a backbone. In rASIC3, there is potential for six acidic residues that face the pore entrance. **c**–**f** Representative whole-cell patch-clamp electrophysiology recordings of cASIC1, rASIC3, cASIC1 G429E, and rASIC3 E435G are (*V*_hold_ = −70 mV). Test solutions were administered for 5 s before returning to normal external solution. ASIC subtype activation was achieve using pH 6.6 and 1 mM CaCl_2_ activating solution and moderate activation with nominal CaCl_2_ (~0 mM, open bar) concentrations in the conditioning pH 7.4. Scale bars are in picoAmperes (pA) and seconds (s). Percent of pH 6.6 peak current amplitude are summarized for cASIC1 (2.8 ± 1.1%, *n* = 13), cASIC1 (G429E) (13 ± 1.1%, *n* = 8), rASIC3 (73 ± 3.1%, *n* = 11), and rASIC3 (E435G) (28 ± 5.6%, *n* = 6). The current from cASIC1 G429E (*p* = 0.0008) and rASIC3 E435G (*p* = 0.00007) were significantly different from the respective wild-type controls. Error bars are s.e.m.

If the relief of Ca^2+^ block activation mechanism was behind the gating characteristics of ASIC3^19^, the residues near the channel gate may differ between the ASIC subtypes. We compared the primary sequences between cASIC1 and rASIC3 in the transmembrane segments to pinpoint the residues that were responsible for ASIC distinct Ca^2+^ sensitivities. Interestingly, we identified a residue positioned approximately one alpha-helical turn from the aspartate gate (Asp433 in cASIC1), which corresponds to glycine in cASIC1 (Gly429) but is glutamate in rASIC3 (Glu435) (Fig. 1a, b; Supplementary Fig. 1). Homology modeling of rASIC3 using the closed cASIC1 pore structure^21^ as the template reveals that the rASIC3 Glu435 is located immediately above the channel gate and thus likely has an influence on ion coordination (Fig. 1b). Therefore, we hypothesize that rASIC3 Glu435 contributes to the Ca^2+^ block site that is absent in most ASIC subtypes.

To test this hypothesis, we exposed each ASIC to a control solution of pH 6.6 in the presence of Ca^2+^ as the control response (assigned as 100%) and subsequently to pH 7.4 environment with no Ca^2+^ (Fig. 1c–f). Channel opening as the result of removing Ca^2+^ is a characteristic that identifies a channel as bearing the ASIC3-like phenotype. The conditioning pH of 7.4 was chosen based on the observed lack of activity at this pH for the ASICs tested. cASIC1 responded to pH 6.6 as expected and had little response to the removal of Ca^2+^ (2.8 ± 1.1% of the pH 6.6 peak current amplitude; Fig. 1c and Table 1). In contrast, rASIC3 responded more robustly (73 ± 3.1% of the control response; Fig. 1d and Table 1) when Ca^2+^ was removed, distinguishing the distinct channel opening properties between cASIC1 and rASIC3 in responses to Ca^2+^.

**Table 1:** Summary of Ca^2+^ response in ASIC channels at pH 7.4

Receptor	*I* at 0 mM Ca^2+^ (% of *I*_pH6.6_)	No. of cells
cASIC1	2.8±1.1	13
cASIC1 (G429E)	13±3.1^a^	8
rASIC3	73±5.8	11
rASIC3 (E435G)	28±5.6^b^	6

Statistics performed using Student’s *t-*test. *I*, peak current amplitude

^a^(*p* = 0.0008), compared to cASIC1

^b^(*p* = 0.00007), compared to rASIC3

To test our hypothesis that the glutamates that were an alpha-helical turn away from the gate were responsible for a Ca^2+^-binding site, we generated two mutant ASICs: rASIC3 E435G and cASIC1 G429E. If our hypothesis was correct, rASIC3 E435G would be expected to influence the amplitude of ASIC3 opening in the absence of Ca^2+^, mimicking the cASIC1 properties. Similarly, we would expect the reciprocal mutant cASIC1 G429E to mimic rASIC3. As we expected, the rASIC3 E435G eliminated approximately 62% of the Ca^2+^ sensitivity in rASIC3 (relative current = 28 ± 5.6%; Fig. 1f and Table 1) compared to the rASIC3 wild type (73 ± 3.1%; Fig. 1d and Table 1**)**, resulting in a channel with remarkably reduced currents compared to its wild type and other channels studied (Fig. 1c–f). On the other hand, the point mutation at cASIC1 (G429E) exhibited a noticeable response to the removal of Ca^2+^ (13 ± 1.1%; Fig. 1e and Table 1) compared to the cASIC1 wild type (2.8 ± 1.1%; Fig. 1c and Table 1**)**, indicating an ASIC3-like Ca^2+^ sensitivity afforded by the glutamate substitution.

### The Ca^2+^ influence on rASIC3 is distinct from that of cASIC1

**Fig. 2:**
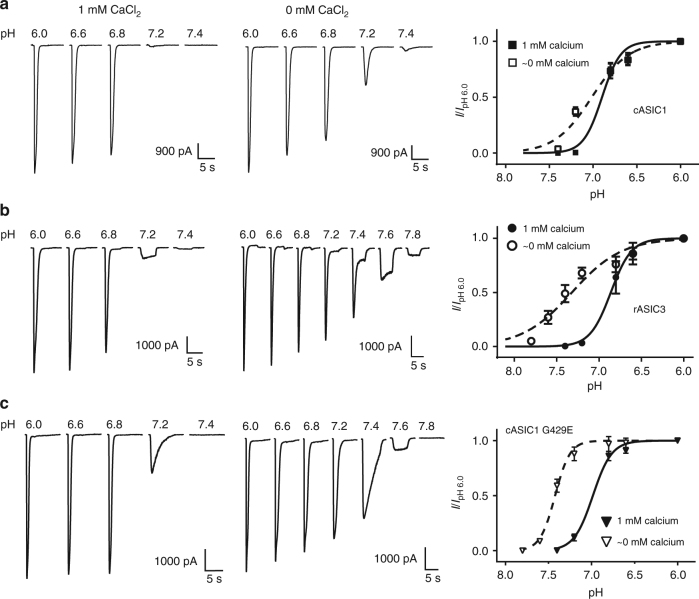
A glutamate for glycine mutation in cASIC1 shifts pH sensitivity to more alkaline values. Whole-cell patch-clamp electrophysiology recordings of cASIC1, rASIC3, and cASIC1 Gly429Glu in transfected CHO-K1 cells (*V*_hold_ = −70 mV) are shown and summarized. Each expressed receptor was exposed to an acidic test solution in the presence or absence of CaCl_2_ for 5 s before returning to pH 8.0 external solution in the presence or absence of CaCl_2_. The summarized data show the response to low pH in the presence of Ca^2+^ (filled symbols) and in the absence of Ca^2+^ (open symbols). **a** cASIC1 had a calculated pH_50_ of 6.90 ± 0.03 with a Hill value of 4.07 ± 0.75 in the presence of Ca^2+^ (*n* = 5 cells). In the absence of Ca^2+^, the pH_50_ was 7.01 ± 0.03 with a Hill value of 2.05 ± 0.23, with both being significantly different from the control (*p* < 0.001, *n* = 5 cells). **b** rASIC3 had a pH_50_ of 6.86 ± 0.02 with a Hill value of 3.54 ± 1.08 in the presence of Ca^2+^ (*n* = 4 cells) compared to a pH_50_ of 7.32 ± 0.04 (*p* < 0.001) and Hill value of 1.48 ± 0.20 (*p* < 0.05) in the absence of Ca^2+^ (*n* = 4 cells). **c** The cASIC1 G429E mutant had a pH_50_ of 6.98 ± 0.02 (*p* < 0.05 compared to wild-type cASIC1) and Hill value of 3.96 ± 0.33 in the presence of Ca^2+^ (*n* = 9) and pH_50_ of 7.42 ± 0.01 and Hill value of 4.99 ± 0.08 in the absence of Ca^2+^ (*n* = 6 cells). Here, the pH_50_ value was significantly different from the calcium response of the mutant (*p* < 0.001) and to the wild-type cASIC1 response under the same conditions (*p* < 0.01). Error bars are s.e.m.

The glutamate residue, rASIC3 Glu435, influenced channel opening following the removal of Ca^2+^ and the corresponding substitution in cASIC1 (G429E) conferred rASIC3-like characteristics. Next, we investigated the influence of this residue on the proton sensitivity. We generated the pH response profiles for the wild-type cASIC1 and rASIC3, along with the cASIC1 G429E mutant. In these experiments, both conditioning and test solutions either had 1 mM CaCl_2_ or no additional CaCl_2_. The cASIC1 pH sensitivity was influenced by the presence of 1 mM Ca^2+^ (Fig. 2a), displaying a pH_50_ of 6.90 ± 0.03 with a Hill value of 4.07 ± 0.75 in 1 mM CaCl_2_ (Table 2, *n* = 5). When cASIC1 was activated in the absence of Ca^2+^ (i.e., at an effective 0 mM CaCl_2_ concentration), the pH_50_ was 7.01 ± 0.03, a more alkaline value, with a Hill coefficient of 2.05 ± 0.23 (Table 2, *n* = 5). Both the low calcium pH_50_ and Hill value were significantly different than the corresponding values in the presence of Ca^2+^ (*p* < 0.001). rASIC3 has an established sensitivity to extracellular Ca^2+19^. Here, we confirmed that rASIC3 became more sensitive to alkaline pH in the absence of Ca^2+^ by showing a pH_50_ value of 7.32 ± 0.04 with a Hill coefficient of 1.48 ± 0.20 (Fig. 2b and Table 2, *n* = 4). The value represents a significant difference (*p* < 0.01) compared to that observed in the presence of Ca^2+^: pH_50_ of 6.86 ± 0.02 with a Hill value of 3.54 ± 1.08 (Fig. 2b and Table 2, *n* = 4).

**Table 2:** Summary of the pH activation for cASIC1, rASIC3 and cASIC1 G429E in the absence and presence of Ca^2+^

Channel	pH_50_	Hill value (*n*_H_)	No. of cells
cASIC1 + Ca^2+^	6.90±0.03	4.07±0.75	5
cASIC1 − Ca^2+^	7.01±0.03^a^	2.05±0.23^a^	5
rASIC3 + Ca^2+^	6.86±0.02	3.54±1.08	4
rASIC3 − Ca^2+^	7.32±0.04^a^	1.48±0.20^b^	4
cASIC1 (G429E) + Ca^2+^	6.98±0.02^c^	3.96±0.33	9
cASIC1 (G429E) − Ca^2+^	7.42±0.01^a,d^	4.99±0.08	6

+ Ca^2+^ and − Ca^2+^ denote conditioning and test solutions include 1 mM or ~0 mM CaCl_2_, respectively

^a^Indicates significantly different compared to the + Ca^2+^ response (*p* < 0.001)

^b^Indicates significantly different compared to the + Ca^2+^ response (*p* < 0.05)

^c^Indicates statistically significant from the wild-type cASIC1 response (*p* < 0.05)

^d^Indicates statistically significant from the wild-type cASIC1 response (*p* < 0.01)

The pH_50_ values are significantly different between the wild-type cASIC1 and G429E mutant channels in the presence of Ca^2+^ (6.90 ± 0.03 in WT vs 6.98 ± 0.02 in G429E; *p* < 0.05, unpaired *t*-test). In the absence of Ca^2+^, pH_50_ in the mutant G429E was shifted to more alkaline (7.42) than that in wild type (7.01) (*p* < 0.01; Fig. 2c and Table 2), which is consistent with gain of the Ca^2+^ block phenotype. The pH sensitivity of cASIC1 G429E therefore resembled that of rASIC3 as shown previously^19^ and here. Taken together, the above results demonstrated the critical role of Glu435 for the rASIC3 proton sensitivity and channel opening after Ca^2+^ removal. The rASIC3 E435G mutation was not subjected to of pH response profile studies. Only 44% (7/16 cells) of GFP-positive cells had noticeable currents elicited by pH 6.6. The average current is 134 ± 41 pA in rASIC3 E435G compared to 5397 ± 706 pA in wild-type rASIC3. These data suggest that the single mutation at ASIC3 may impair channel insertion into the membrane or the gating mechanism. Poor functional expression prohibited us from achieving stable recordings required for pH response studies.

### MD simulations reveal an ASIC3 high-affinity pore Ca^2+^ site

**Fig. 3:**
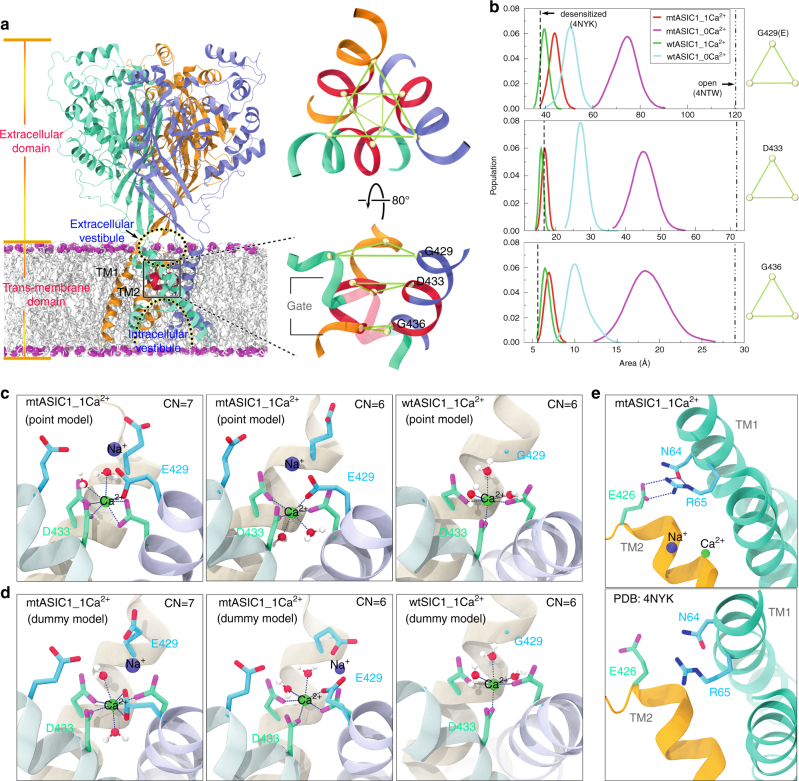
Molecular dynamics simulations reveal a stable Ca^2+^-binding site in the ASIC channels. **a** cASIC1 homotrimer embedded in the POPE lipid bilayer and close-up view of the channel pore. The lipid phosphorus atoms are showed as magenta spheres and the three protein subunits are represented as cyan, orange and ice blue ribbons, respectively, with the pore gate highlighted in red. The extracellular and intracellular vestibules are indicated by dotted lines. The Cα atoms of the residues at positions 429 (target mutation site), 433, and 436 are displayed as spheres. **b** Probability distribution of the area of the triangle formed by the Cα atoms of symmetry-related Gly429 (or Glu429 in the mutant systems) (upper), Asp433 (middle), and Gly436 (lower). The red and magenta curves correspond to the cASIC1 G429E systems with 1 Ca^2+^ (mtASIC1_1Ca^2+^) and without Ca^2+^ (mtASIC1_0Ca^2+^) bound, respectively, and the green and cyan curves denote the wild-type systems with 1 Ca^2+^ (wtASIC1_1Ca^2+^) and without Ca^2+^ (wtASIC1_0Ca^2+^), respectively. The dashed and dashed-dotted lines denote the area values calculated from the crystal structures in desensitized and wide open states (PDB codes: 4NYK & 4NTW), respectively. **c**, **d** The Ca^2+^ block site identified at the pore entrance with the Ca^2+^ point charge (**c**) and dummy atom models (**d**). The Ca^2+^ in the mutant channel (mtASIC1_1Ca^2+^) forms both pentagonal bi-pyramidal and octahedral coordination (first and second panels), whereas the Ca^2+^ binding in the wild channel (wtASIC1_1Ca^2+^) assumes an exclusive octahedral geometry (third panel). The residues Glu429 and Asp33 and water molecules coordinating Ca^2+^ are represented in stick and ball-and-stick models, respectively, and the Cα atom of Gly429 in the wild-type system is shown as a sphere. The coordination number (CN) is indicated at the upper-right corner of each panel. For clarity, the dummy complex in **d** has been shown as a Lennard–Jones sphere as in **c**. Statistical analysis of the coordination bonds and angles is reported in Supplementary Table 2. **e** Inter-subunit ionic interactions of Glu426 in TM2 and Arg65 in TM1 stabilizing a putative intermediate state of the G429E mutant

Based on our experimental mutagenesis data, we propose there exists a Ca^2+^ block (binding) site at the beginning of the transmembrane segments in rASIC3 that subtly defines its unique Ca^2+^ sensitivity. To shed additional light on this mechanism, we performed all-atom molecular dynamics (MD) simulations on the cASIC1 G429E mutant and wild-type channels in an explicit solvent and membrane environment with and without Ca^2+^ at the channel pore mouth (Fig. 3a) (see Methods). cASIC1 G429E served as a mimic of ASIC3, given the high sequence identity among the ASIC subtypes in the pore-lining TM2 (Supplementary Fig. 1). Starting from the cASIC1 desensitized state structure, our simulations demonstrated that release of Ca^2+^ led to widening of the channel pore, which is much more remarkable for the G429E mutant than for the wide type (Fig. 3b). Using the Asp433 Cα atom for quantitative estimates, the peak value of the area for the mutant channel increased by ~167% upon removal of Ca^2+^, while the corresponding percent increase was only ~68% for the wild type (middle panel, Fig. 3b). The data thus implied that ASIC3 has a distinct gating mechanism from ASIC1 in response to varying Ca^2+^ conditions. The mutant channel pore expansion resulted from an iris-like rotation of TM1 and TM2a (i.e., the extracellular portion of TM2) upon removing Ca^2+^ (Supplementary Fig. 2). To clarify whether charge or size of the side chain at this position is more important for channel opening, we performed a set of simulations on the cASIC1 G429F mutant. Using the wild-type cASIC1 as a reference, it is evident that the G429E channel opened more prominently than the G429F did (Supplementary Fig. 3), suggesting charge plays much more important role in channel opening than size of the side chain at this position. Our observations imply that stronger electrostatic repulsion at the ASIC3 Ca^2+^-binding site enables it to open more readily than ASIC1 does upon removal of Ca^2+^.

Using two distinct ion models representing the Ca^2+^ (Supplementary Fig. 4), we identified a consistent Ca^2+^-binding site at the interface of the channel gate and the extracellular vestibule in either of the cASIC1 G429E and wild-type systems (Fig. 3c, d). The Ca^2+^ ion in the mutant channel formed a pentagonal bi-pyramidal coordination (involving 7 ligands) or an octahedral coordination (involving 6 ligands) with the conserved gating residue Asp433 on each subunit, Glu429 on one subunit, and two water molecules (first and middle panels, Fig. 3c, d). The coordination number (CN) of Ca^2+^ for the Ca^2+^-binding proteins usually ranges from 6 to 8, and for CN > 6 the bi-dentate coordination is often implicated (Supplementary Fig. 5a)^24^, as observed herein. The Ca^2+^-binding site revealed here is reminiscent of the voltage-gated calcium channel Ca_v_Ab^25^ and the Ca^2+^-selective transient receptor potential (TRP) channel TRPV6^26^ that also exploit a cluster of four carboxylate groups for binding Ca^2+^ (Supplementary Fig. 5b). Interestingly, the wild-type channel trapped the Ca^2+^ ion exclusively in an octahedral form, wherein each subunit Asp433 contributes one ligand in addition to three water molecules (last panels, Fig. 3c, d). Due to the involvement of one more acidic residues (Glu429), the binding affinity of the mutant (G429E) channel to the Ca^2+^ ion was estimated to be ~20% higher compared to the wild-type channel with the dummy atom model (Supplementary Fig. 6), or ~40% higher with the point charge model (Supplementary Note 1). The high-affinity Ca^2+^-binding site identified here thus accounts for the unique Ca^2+^ sensitivity of ASIC3^19^. In the meanwhile, we note the used Ca^2+^ models might face difficulty in treating bi-dentate versus mono-dentate coordination in the current simulations employed the classic, non-polarizable force fields (Methods)^27,28^. The use of non-polarizable force fields also tends to overestimate the binding affinity to Ca^2+^; however, the results are reasonable as a relative measure^29–31^.

### The G429E mutant illuminates the role of Arg-Glu pair gating

Acting like a plug, the single bound Ca^2+^ impeded water flux through the gate (Supplementary Fig. 2). Interestingly, we discovered a Na^+^ primed for inward entry wandering just above the bound Ca^2+^ ion in the simulations of cASIC1 G429E mutant (first and middle panels, Fig. 3c, d; Supplementary Fig. 2a). Moreover, the bottom of the extracellular vestibule was more hydrated in the Ca^2+^-free channels than in the Ca^2+^-bound counterparts (lower panels, Supplementary Fig. 2). A continuous water file occasionally formed through the entire channel in the Ca^2+^-free mutant system (with ~0.10% probability). Yet, the Na^+^ inside the extracellular vestibule failed to diffuse into the intracellular vestibule through the channel gate (lower panel, Supplementary Fig. 2b), which is likely due to the incomplete opening of the channel from the current microsecond-scale simulations (Fig. 3b).

Further inspection of the simulation trajectories revealed an expanded, non-conductive, intermediate state between the closed and open states in the mutant channel (Fig. 3b; Supplementary Fig. 2b). Transmembrane helical displacements (upper panel, Supplementary Fig. 2b) made space for the TM2 Glu426 side chain rotation to form ionic and hydrogen-bonding interactions with Arg65 from an adjacent subunit immediately preceding TM1 (upper panel, Fig. 3e**)**. The same inter-subunit interactions are disfavorable in the desensitized state due to steric clashes (lower panel, Fig. 3e) or collapsed in the full opening conformation (Supplementary Fig. 7a). Scrutiny of available ASIC primary sequences showed that the Arg-Glu residue pair is highly conserved amongst different subtypes except ASIC4 (Supplementary Fig. 2). Based on these observations, we propose that the Arg-Glu pair likely plays a role in the channel gating transition by alternative engagement and disengagement in response to extracellular Ca^2+^/H^+^ concentration changes.

### Titration simulations reveal H^+^/Ca^2+^ gating site competition

In the above simulations, the gating residue Asp433 was assumed to be in a deprotonated state (i.e., at high pH environment). To explore the binding completion between H^+^ and Ca^2+^ at the gating site, we carried out a series of MD simulations on the cASIC1 wild type and G429E mutant (a rASIC3 mimic) channels under various protonation conditions to probe the impact of protonation at the Asp433 ring on Ca^2+^ affinity (Supplementary Table 1). Herein, the relative binding affinity was estimated by the end-point MM-GBSA (molecular mechanics/generalized born surface area) approach^32,33^ (Methods section). For the mutant system, single and double protonation at the Asp433 cluster resulted in negligible changes in Ca^2+^-binding affinity (Supplementary Fig. 6), which is due to a surplus of coordinating carboxylates available at the binding site (Supplementary Fig. 8a). In contrast, triple neutralization at Asp433 reduced the Ca^2+^-binding affinity considerably and opened the channel pore slightly (Supplementary Fig. 8b). Presumably, further protonation at the gating site (i.e., increasing H^+^ concentration) would effectively destabilize Ca^2+^ and eventually eliminate Ca^2+^ blockade. The wild-type system exhibited a similar trend, but required less protons to destabilize Ca^2+^ binding (Supplementary Figs. 6 and 8c), which is consistent with the proton dose–response experiments suggesting a more alkaline pH_50_ with cASIC1 than that with rASIC3 and cASIC1 G429E in the presence of Ca^2+^ (Fig. 2 and Table 2). In addition, the cASIC1 wild-type channel pore opened little as a result of protonation at Asp433 (Supplementary Fig. 8d), as compared to the mutant (Supplementary Fig. 8b). Hence, the protonation simulations here provided direct evidence to support the previous argument by Immke and McCleskey^19^ that multiple protons and one Ca^2+^ compete for the same gating site above the channel pore. In the meanwhile, we noted that the difference of Ca^2+^ affinities might be underestimated from our conventional simulations with an invariable protonation state initially assigned for the titratable sites^34^. To relieve this problem, the recently developed pH titration MD (pHtMD) protocol^35^ could be a desirable choice for further studying pH-dependent effect on channel gating. Yet, it would not qualitatively influence the overall trend herein.

## Discussion

Here, we identify a single, titratable, high-affinity Ca^2+^-binding site above the channel gate in rat ASIC3 and engineer this site within cASIC1 G429E mutant receptor. This site implicates four acidic residues, including three symmetry-related aspartate residues and one glutamate residue from either subunit (Fig. 3c, d). Previous studies^19^ by Immke and McCleskey suggested that the relief of Ca^2+^ block was the dominant activation mechanism in ASIC3. Since then, this model has been controversial, mainly due to lack of the structural and dynamic characterization of the putative Ca^2+^-blocking site. Electrophysiology compared the pH sensitivities between the wild-type cASIC1, rASIC3, and cASIC1 (G429E) mutant channels. The pH sensitivities of the rASIC3 and the mutant channel were remarkably similar, with pH sensitivity shifting to more alkaline pH values in the absence of Ca^2+^ (Fig. 2 and Table 2). This alkaline shift suggested that the Ca^2+^ site above the ASIC3 and cASIC1 G429E pore is the dominant influencing Ca^2+^ site for pH sensitivity. Furthermore, the introduction of the ring of glutamates in substitution of the glycine residue into cASIC1 resulted in opening at pH 7.4, a condition where most channels are presumed to be closed, and a gating characteristic of ASIC3 (Fig. 1). It appears that a single point mutation is sufficient to convey rASIC3 Ca^2+^ sensitivity properties to cASIC1.

Introducing the cASIC1 G429E mutation has a pH_50_ value between that of cASIC1 and rASIC3 and removing Ca^2+^ results in a significant alkaline shift in pH_50_, similar to ASIC3. This suggests that the Gly to Glu change near the channel pore greatly influences pH sensitivity and channel gating. The pH_50_ shift to more ASIC3-like properties by introducing the mutant supports our contention that the original relief of Ca^2+^ block model is relevant here due to the presence of a dominant Ca^2+^-binding site in ASIC3. A reduction in Hill value shows that cooperativity between binding sites has been affected by the removal of Ca^2+^ and introduction of the point mutation^36^. We observed that when cASIC1 and rASIC3 were activated in the absence of Ca^2+^, there was a reduction in Hill values, suggesting that the proton-binding sites moved toward non-cooperativity. In the cASIC1 G429E mutant, the Hill value did not change significantly, suggesting that cooperativity remained the same. MacLean and Jayaraman^37^ discussed how the Hill slope in ASICs accounts for many fundamental characteristics of the channel (gating, agonist-binding, desensitization, etc.). Based on the data presented here, we conclude that the mutation has introduced proton-binding site cooperativity that does not change when Ca^2+^ is removed from external solution. Further study is needed to demonstrate how this site affects specific ASIC gating properties, such as activation and deactivation kinetics.

We contend that rASIC3 Glu435 contributes to a higher-affinity Ca^2+^-binding site at the extracellular mouth of the channel. Conversely, we have provided evidence to suggest that a weak Ca^2+^-binding site is present at the pore gate in other ASICs. The gating aspartate residue (cASIC1 Asp433) is highly conserved across all of ASICs (Supplementary Fig. 1), and presumably could alone constitute a moderate affinity-binding site for various divalent cations that controls gating in some specific ASICs other than the ASIC3 subtype. In the same region, electron density features were found in cASIC1 protein crystals soaked with the cation cesium (Cs^+^)^21^. One of the proposed cesium densities appeared to coordinate with the carboxylates of cASIC1 D433. What was not seen in the protein electron density map were other additional contributors to this coordination, unlike the coordination observed with the second cesium electron density. The second cesium ion coordinated with the carboxylates of D433 and the backbone oxygen atoms of G432, in a trigonal antiprism pattern. Coordinating with cesium provides evidence that these residues coordinate with cations, in at least a weak-binding site, and suggests that other cations (such as the divalent cation Ca^2+^) may bind here. Furthermore, the presence of a weak Ca^2+^-binding site at the pore gate may provide an explanation for potentiation of the currents in mouse ASIC1a^38^ and fish ASIC1^16^ by decreasing the concentrations of external Zn^2+^ and Ca^2+^. Interestingly, the glutamate residue (Glu435 in rASIC3) is only conserved among the ASIC3 subtype, whereas the equivalent position is occupied by glycine or alanine for the other ASIC subtypes (Supplementary Fig. 1). Therefore, the gating mechanism revealed herein is likely to be broadly conserved across diverse ASIC3 members in different species. However, we cannot rule out species specific differences in channel activity, as seen with the human ASIC3 sensitivity to alkaline pH^39^, a characteristic absent in rat ASIC3.

The stoichiometry of four carboxylates and one Ca^2+^ at the gating site revealed here is consistent with the previous experimental and theoretical estimates^19^ suggesting that the reaction orders for repriming from desensitization are as high as 4 for H^+^ at a relatively low Ca^2+^ concentration of 1 mM (approximate to that in the simulation) and always 1 for Ca^2+^. However, the Immke–McCleskey model suggested Ca^2+^/H^+^ site catalysis fails to trigger a conformational change; in other words, these channels should be constitutively active (open) upon removal of Ca^2+^ ^18,19^. In contrast, our simulations of Ca^2+^-bound and Ca^2+^-free channels demonstrated that considerable conformational changes occurred (Fig. 3b; Supplementary Fig. 2). We mapped the identified Ca^2+^ block site alongside Ca^2+^ onto the MitTx-bound open ASIC1 channel^22^ to determine whether the open channel could be occluded by Ca^2+^. The acidic residue ring is out of reach of coordinating Ca^2+^ in the open state (Supplementary Fig. 7b), thereby suggesting a moderate, if not significant, conformational change must be involved during the gating process.

Though simple, the model of a single Ca^2+^ block site above could explain both the inhibition of acid-sensing ion channel conductance and the modulation of its H^+^ sensitivity by Ca^2+^. However, Paukert et al.^18^ predicted the existence of a second Ca^2+^-binding site that allosterically modulates channel opening, given the observation that disruption of the identified Ca^2+^-binding sites (Glu425 and Asp432) in rat ASIC1a (rASIC1a) by single (E425G and D432C) or combined substitutions did not open the channels constitutively. In light of the crystallographic and computational data, we contend that a re-interpretation of the previous experimental data is warranted. The rASIC1a Glu425 (Glu426 in cASIC1) is very unlikely to comprise an additional Ca^2+^ binding/modulating site in that: (i) it is exposed to the outside of the extracellular vestibule in our simulations and the reported cASIC1 structures^21–23,40^; (ii) the Glu426 ring diameter is large (22~30 Å) in each structure; and (iii) Glu426 lacks partners to coordinate/stabilize Ca^2+^. Instead, the rASIC1a E425G mutation may result in disengagement with Arg64 (Arg65 in cASIC1) in the intermediate state as indicated, thereby facilitating pore opening and destabilization of Ca^2+^ binding, especially alongside D432C replacement. Additionally, the D432C mutation is not able to completely destroy the Ca^2+^ block site as the cysteine itself can contribute a ligand via the cysteine sulfhydryl^24^. In view of the spatial proximity of the trapped Ca^2+^ to the Asp433 side chains (Fig. 3c, d), a low-affinity Ca^2+^ block site can possibly form by exploiting two, if not all, of the three cysteines. The above analyses thereby exclude the possibility of Glu425 as the allosteric site in rASIC1a, and account for the reduction of Ca^2+^ block by either the single substitutions and the abolishment of Ca^2+^ block by the double mutation (a combined effect)^18^. Furthermore, the observation that the rASIC1a double mutant retains the principal gating characteristics of the wild type (i.e., activation by H^+^ and modulation of H^+^ sensitivity by Ca^2+^)^18^ suggests the possibility for ASIC1 to have Ca^2+^/H^+^ binding sites in the extracellular domain that allosterically mediate functional conformational changes.

The crystal structures and the experiments of cASIC1^21,40^ have revealed a prominent acidic pocket located in the extracellular domain, where there are several pairs of close carboxyl–carboxylate interactions between aspartate and glutamate residues (such as Asp238–Asp350, Glu239–Asp346 and Glu220–Asp408). This acidic pocket was proposed to be engaged in the H^+^/Ca^2+^ sensing and the associated allosteric control of channel activation^21,40^. Sequence analysis shows that the equivalent residue to cASIC1 Asp346 is a serine in rASIC3, the only difference in the acidic pocket composition between the two channels. To test the hypothesis that the residues in the acidic pocket function as the H^+^/Ca^2+^ allosteric site, we additionally performed two sets of simulations (S13, S14, Supplementary Table 1): in S13, Asp346 is protonated; in S14, Asp346 is replaced with Ser. Within several microsecond timescale, we observed no detectable influence of either protonation or mutation on the affinity and mode of Ca^2+^ binding within the channel pore (Supplementary Fig. 6; Supplementary Table 2). Given the large separation (~55 Å) between the putative allosteric site and the identified gating site in the transmembrane domain, the actual remote communication between the two sites generally occurs on the millisecond to second timescale, which is unreachable for the present conventional simulations. Future experiments and enhanced dynamics simulations are needed to answer this question. Hence, we do not exclude the importance of the allosteric model in understanding ASIC gating mechanisms. For ASIC1, we noticed that there are increasing data supporting an allosteric gating mechanism, partly because the Ca^2+^ block model cannot fully account for the observed experimental phenomena. Concerning the ASIC3 studied here, the relief of high-affinity Ca^2+^ block plus an accompanying local conformational change is sufficient to account for its activation gating. Therefore, gating models for different ASIC subtypes or even the same subtype family may differ, having significant implications in other fields of study (such as pharmacology and drug design).

The channel-binding site for Ca^2+^ is strengthened by the presence of additional acidic residues near the channel mouth. The inclusion of the Gly to Glu mutation at the cASIC1 position 429 and the converse in rASIC3 may be key for the Ca^2+^ block site in the ASICs studied. A similar Ca^2+^ sensitivity was observed in marine ASIC, where a reduction in activating solution pH and Ca^2+^ concentration resulted in a shift towards more alkaline pH in the activation profile^16^. From this study, there was a significant finding in support of our proposal that there are weak Ca^2+^ block sites in non-ASIC3 subtypes. In the marine ASIC study, Ca^2+^ blocked low pH generated current in a voltage-dependent manner at higher concentrations that what are needed for gating, which suggests that there is a weak Ca^2+^ block site within the electric field, or channel pore^16^. The marine ASIC study highlights the need to keep under consideration that differences exists between the ASIC subtypes.

In summary, the combined electrophysiological and computational results suggest that the Ca^2+^-block site at the entry of the ASIC3 channel pore is the result of a single acidic residue one alpha-helical turn away from the canonical ASIC gate. The data here confirm the presence of a high-affinity Ca^2+^-binding site and reveal for the first time the Ca^2+^-binding mode at this block site. Future studies, and development of novel therapeutics targeting ASICs, will be needed to account for the unique differences in ASIC subtype gating and calcium sensitivity.

## Methods

### Chemicals

All reagents were purchased from Sigma-Aldrich. The external pH was adjusted using *N*-methyl-d-glucamine.

### Cell culture and ASIC expression

Full-length chicken ASIC1 and rat ASIC3 were subcloned into a vector that attached an enhanced green fluorescent protein to the amino terminus^41^. Site-directed mutagenesis for cASIC1 G429E and rASIC3 E435G were made using the QuikChange Lightning Site-Directed Mutagenesis kit, according to manufacturer’s instructions (Agilent Technologies). Each ASIC construct was transiently transfected into Chinese hamster ovarian (CHO-K1, ATCC CCL-61) cells for electrophysiological study using Lipofectamine LTX (Life Technologies, according to manufacturer’s guidelines), as previously reported^41^. Briefly, cells were plated on 9×9 mm glass coverslips in 35 mm cell culture dishes in completed growth media (Ham’s F-12 Nutrient Mixture, 10% fetal bovine serum, and 5% penicillin/streptomycin, from ThermoFisher). After 24 h of incubation, transfection reactions were generated using serum-free media (500 μL) and was placed in sterile microcentrifuge tubes under a laminar flow hood. To each tube, 1 μg ASIC plasmid DNA was added followed by 4 μL of Lipofectamine LTX reagent. Reactions were tapped gently to mix and allowed to incubate for 20 min at room temperature. Next, media was removed from the cell culture dishes and replaced with fresh complete growth media (1.5 mL). Each transfection reaction mix was applied to separate culture dishes, bringing the final concentration to 2 mL. Transfected cells were allowed to grow in a 37 °C/5% CO_2_ incubator for 18–24 prior to electrophysiological assay. Only cells that exhibited fluorescence were chosen for electrophysiological study.

### Whole-cell patch-clamp electrophysiology

Patch pipettes were pulled from borosilicate glass and fire-polished with a final resistance 5–7 MΩ (Flaming/Brown, P-87/PC, Sutter Instrument CO., Novato, CA). Whole-cell patch-clamp electrophysiology was performed on an inverted fluorescent microscope fitted with a FITC fluorescence filter (Nikon) that was equipped with an Axopatch 200B patch-clamp amplifier or an upright microscope fitted with a FITC fluorescence filter (Nikon) equipped with a Axopatch 200 B patch-clamp amplifier (Molecular Devices) using pClamp10 data acquisition software. External solutions were applied at room temperature and mimicked the extracellular and intracellular cellular compartment. Extracellular bath and test solutions consisted of (in mM): NaCl (150), KCl (5), MES (5), HEPES (5), with pH adjusted using HCl or *N*-methyl-d-glucamine (NMDG). Additionally, intracellular solutions contained (in mM): KCl (100), MgCl_2_ (5), EGTA (10), HEPES (40), and NaCl (5) at pH 7.35. The extracellular base solution was used for all other solutions and adjusted to the specified pH. Calcium was added at a concentration of 1 mM in calcium-rich solutions and omitted from those mimicking calcium-depleted solutions (nominal calcium). Solution exchange was obtained using arrays of microcapillary tubes arranged perpendicularly to the patch cell using digitally controlled PTFE solenoid or pinch valves with a ValveLink8.2 controller (AutoMate Scientific) or a gravity driven Y-tube solution delivery system.

### Homology model generation

A three-dimensional model was generated using the cASIC1 desensitized crystal structure (PDB code 4NYK)^42^ using SWISS-MODEL webservers^43^. Trimeric arrangements for both cASIC1 and resulting rASIC3 models were generated using Pymol molecular visualization system^44^ with a symmetry command.

### Electrophysiology data analysis

Peak current amplitudes of test solutions were normalized against a control pH response in each experiment. Statistical significance was determined using unpaired Student’s *t*-test or* F*-test, where appropriate. pH response profiles were generated for wild-type cASIC1, rASIC3, and cASIC1 G429E mutant receptors in the presence and absence of calcium to determine the pH that elicits half of the maximal response, or pH_50_ by fitting with a logarithmic equation. For pH response profiles, pH_50_ and Hill values, significance was determined using the sum of least-square *F* test (GraphPad Prism 6). For response to removal of calcium at pH 7.4, upaired Student’s *t*-test was used for comparison.

### Computational system setup

Since no rASIC3 structure is available, we chose the minimally functional construct (residues 46–451) of the chicken ASIC1 (cASIC1) determined in a low pH desensitized state (PDB code: 4NYK^21^) as a starting point for all the simulations herein. The channel pore of this desensitized structure is closed and assumes considerable conformational similarity to that of the resting, closed state channel^21,45,46^. Given that this work is mainly focused on the transmembrane domain, the used structure is suitable for the simulation aim, though the major difference between the two states in the extracellular domain^20,21,46^. To test our experimental hypothesis, four simulation systems, i.e., the cASIC1 G429E mutant with/without Ca^2+^ and the wild type with/without Ca^2+^, were built based on the same procedures. The bound Cl^−^ and water molecules in the crystal structure were retained. To generate the Ca^2+^-containing systems, 1 Ca^2+^ ion was placed at the mouth of channel pore (near the external vestibule base) by reference to the position of Cs^+^ ion at site 2 in the Cs^2+^-soaked crystals (PDB code: 3IJ4)^21^. The principal axis of the trimeric cASIC1 was pre-aligned along the *z*-axis (i.e., membrane normal) through the orient plug-in included with VMD^47^. The construction of the channel/membrane systems was then facilitated using the on-line tool CHARMM-GUI Membrane Builder^48^. The disulfide bridges rich in the extracellular domain were explicitly taken into account. The protonation states of the ionizable residues outside of the transmembrane domain were predicted using the web server H++ ^49^, followed by visual inspection. For the symmetry-related Asp433 ring inside the channel pore, four different protonation conditions were considered as described below. The replacement scheme was employed to pack the 1-Palmitoyl-2-oleoyl-*sn*-glycero-3-phosphoethanolamine (POPE) lipid bilayer around the transmembrane zone of the channels. The dimensions of the membrane plane (*x*–*y*) were set to be 118×118 Å, and the water layer thickness from either of the solute boundaries along the *z*-axis be 20 Å. Na^+^ and Cl^−^ were added to yield a physiological salt solution of 150 mM. The above settings resulted in a simulation box of ~118×118×153 Å^3^, a total of 207,000 atoms and a Ca^2+^ concentration of ~0.8 mM for each system.

### Molecular mechanics force fields

Accurate modeling of metal–ion-containing biological systems has been a nontrivial issue in computational chemistry^28,50^. The metal ions are generally represented as a single charged sphere (point charge model) and this model has been widely used in combination with the additive, non-polarizable biomolecular force fields (FF) (such as CHARMM, AMBER, and GROMOS)^51–53^. Despite continuous improvements, the non-polarizable FFs and the non-bonded ion parameter still face limitations in accurately characterizing the ion-ligand interactions due to insufficient inclusion of quantum effects, especially at the enzymatic centers^28,50^. Polarizable FFs and quantum mechanical description of the metal centers provide a more rigorous physical framework, however, the high computational cost make them untenable with increasing system size (like the membrane system herein), particularly in free energy calculations that require extensive sampling of conformations^54^. The alternative strategy, viz., the cationic dummy atom model (a.k.a. multisite ion model)^55^, provides a promising balance between the computational accuracy and efficiency. In this approach, the metal site is represented by a set of dummy atoms connected around a central atom (Supplementary Fig. 4a). By placing the fractional charges between the metal core and surrounding residues, this model has the advantage of mimicking the nature of the covalent bond, thereby offering a more sophisticated electrostatic description. In addition to the traditional point charge model^56^, we resorted to the Ca^2+^ dummy model recently developed by Sept lab^55^ to treat the Ca^2+^-binding interactions with the protein, as this model was shown to achieve remarkable improvements in both coordination and free energy calculations compared to the spherical ions^55^. The Ca^2+^ point charge and dummy models were here accommodated with the CHARMM and AMBER FFs, respectively. Consequently, we have applied two different sets of all-atom FFs for the treatment of the simulations systems. In detail, the CHARMM36 (the July 2016 update) FFs were used for the solutes (proteins and lipids) and all ions (Na^+^, Cl^−^ and Ca^2+^)^57^; the latest AMBER ff14SBonlysc^58^ and lipid17 FFs^59^ were selected for the proteins and lipids, respectively, and the Joung-Cheatham ion parameter sets^60^ for Na^+^ and Cl^−^. In addition, the water was described by the TIP3P model^61^.

### Molecular dynamics simulations

The simulations with the CHARMM FF (plus Ca^2+^ spherical model) and the AMBER FF (plus Ca^2+^ dummy model) were performed using the package NAMD (v2.10)^62^ and the GPU accelerated version of AMBER16 engine PMEMD^63^, respectively. The format conversion of the CHARMM-GUI generated lipids into the AMBER-style lipids was facilitated via the charmmlipid2amber.py script within the AmberTool16^63^. The input parameters were kept as consistent as possible between the two simulation programs. The periodic boundary conditions were applied in all of the simulations. The short-range non-bonded interactions were truncated at 12.5 Å, with the smoothing function switched on from 10 Å, and the long-range electrostatics were calculated via the particle mesh Eward summation method^64^ with a grid spacing of 1 Å. The covalent bonds involving hydrogens were constrained employing the SHAKE algorithm^65^. The time step of integration was set to 1 fs during the minimization and equilibration stages and 2 fs for the production runs. The Langevin thermostat was used to maintain the temperature at 303.15 K, with a damping coefficient of 1 ps^−1^. The pressure was kept constant at 1.013 bar by the Nosé-Hoover Langevin piston with an oscillation period of 100 ps (in NAMD), or by the Monte Carlo barostat with a relaxation time of 2 ps (in AMBER). The trajectory snapshots were recorded at 10-ps intervals. The channel/membrane systems underwent a sequence of steps to reach stability following the collective variable-based equilibration strategy implemented in NAMD^33^. In brief, three different types of restraints were simultaneously imposed and gradually released: the planar restraint is used to keep the lipid head groups on the *x*–*y* plane that is perpendicular to the membrane normal; the root-mean-square displacement (RMSD)-based restraint is used to maintain the global conformation of the protein; and the dihedral restraint is applied to restrain the local backbone configuration of the protein. Each system was then further equilibrated 150 ns, following a production run in the isobaric–isothermal (NPT) ensemble that reaches 1000 ns. Moreover, in evaluating the effect of increasing protons on the binding of Ca^2+^ (i.e., mimicking pH titration), a set of MD simulations on the mutant and wild-type channels were carried out under different protonation circumstances, wherein the symmetry-related Asp433 cluster was singly, doubly or triply protonated, as compared to the fully charged state applied above. A summary of all the simulations reported in this study is presented in Supplementary Table 1.

### Analyses of simulation trajectories

The last 300 ns of the simulation trajectories were extracted for the following analyses. In detail, the binding affinities of Ca^2+^ to the protein were estimated by the MM-GBSA (Molecular Mechanics-Generalized Born Surface Area) approach^32,33^. Compared to MM-PBSA (molecular mechanics-Poisson Boltzmann surface area), MM-GBSA is computationally more efficient and has shown to gain comparable or even better accuracy in predicting the relative affinities^32,66^. Specifically, the five water molecules closest to the Ca^2+^ in each trajectory snapshot were consistently retained and regarded as part of the receptor, given the maximum number of five water molecules involved in coordination with the ion (Fig. 3c, d; Supplementary Fig. 8a, c). This scheme has been applied in our recent works with the nucleases and obtained more reasonable results^67,68^. All of the MM-GBSA calculations were accomplished using the program MMPBSA.py in AmberTools16^63^. To enable the computation of the CHARMM trajectories, the ParmEd program in AmberTools16^63^ was used to convert the CHARMM PSF file to the AMBER-style parameter and topology file. The simulation structures used for visualization and comparison were determined through the cluster analysis with the package VMD (v1.9.2)^47^, as described in our recent studies^67,68^. Moreover, the water density was calculated with the VolMap tool in VMD^47^ and the radius scaling in VolMap was increased to 2 to produce a smoother map. A detailed comparison and an extended discussion of the results with the two different Ca^2+^ models are presented in Supplementary Information.

### Data availability

Data supporting the findings of this manuscript are available from the corresponding authors upon reasonable request.

## Electronic supplementary material

Supplementary Information
