# Supplementary material for: Identification of a unique Ca2+-binding site in rat acid-sensing ion channel 3
Source: Nat Commun. 2018 May 25;9:2082. doi: 10.1038/s41467-018-04424-0 (PMC5970173; doi:10.1038/s41467-018-04424-0)
Supplement: Supplementary file 1 — Supplementary Information [file 41467_2018_4424_MOESM1_ESM.pdf]

Supplementary Information of "Identification of a unique Ca^{2+}-binding site in rat acid-sensing ion channel 3"

Zhicheng Zuo *et al.*

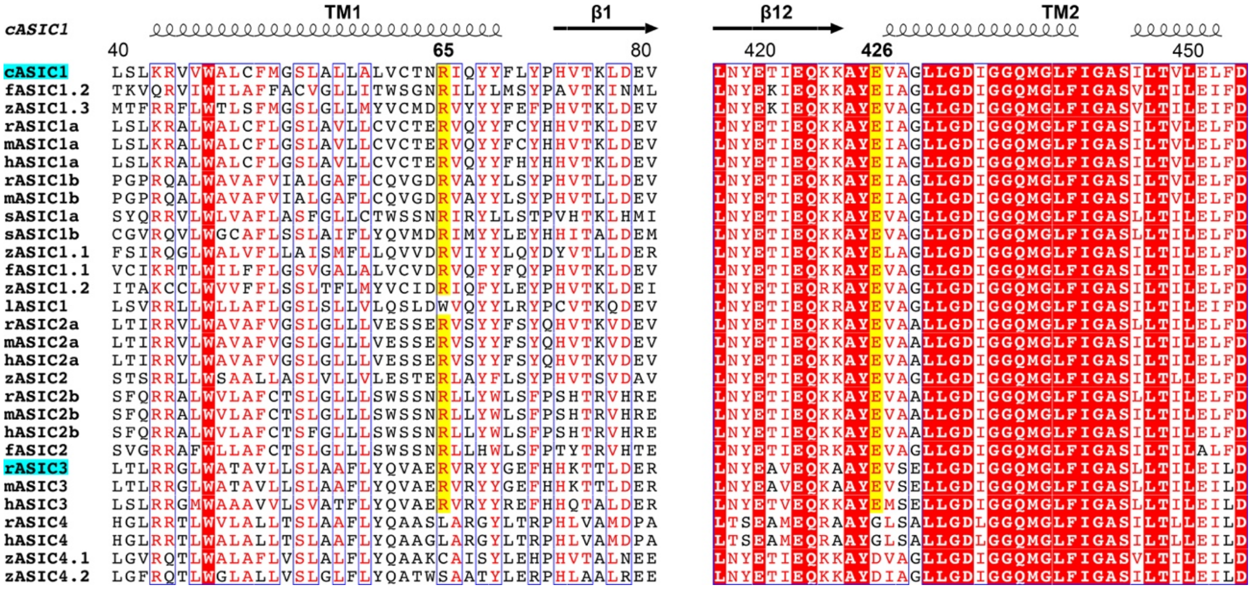

Supplementary Figure 1 | Multiple sequence alignment focusing on the transmembrane domain of ASICs from different species. The primary sequences of ASICs from chicken (cASIC1, GenBank ID: 63054900), toad fish (fASIC1.1, 32264693; fASIC1.2, 32812316; and fASIC2, 32812318), zebra fish (zASIC1.1, 42759896; zASIC1.2, 42759898; zASIC1.3, 47550873; zASIC2, 42759902; zASIC4.1, 42759904; and zASIC4.2, 42759906), rat (rASIC1a, 2039366; rASIC1b, 14799389; rASIC2a, 1280441; rASIC2b, 2815277; rASIC3, 2352949; and rASIC4, 81906594), mouse (mASIC1a, 45433493; mASIC1b, 63003141; mASIC2a, 13811382; mASIC2b, 2815275; and mASIC3, 82582993), human (hASIC1a, 21536349; hASIC2a, 1280439; hASIC2b, 21739677; hASIC3, 3747101; and hASIC4, 8346834), shark (sASIC1a, 63054896; and sASIC1b, 63054898) and lamprey (lASIC1, 63054894) were aligned using CLUSTALW[1]. The alignment was visualized by ESPript[2]. Conserved residues are shown as white text on a red background, and similar residues are as red text on a white background; the cASIC1 Arg65 and Glu426 and the corresponding residues from other members are additionally highlighted in yellow background. The secondary structure of cASIC1 derived from the crystal structure (PDB code: 4NYK[3]) is marked on the top of the sequence alignment.

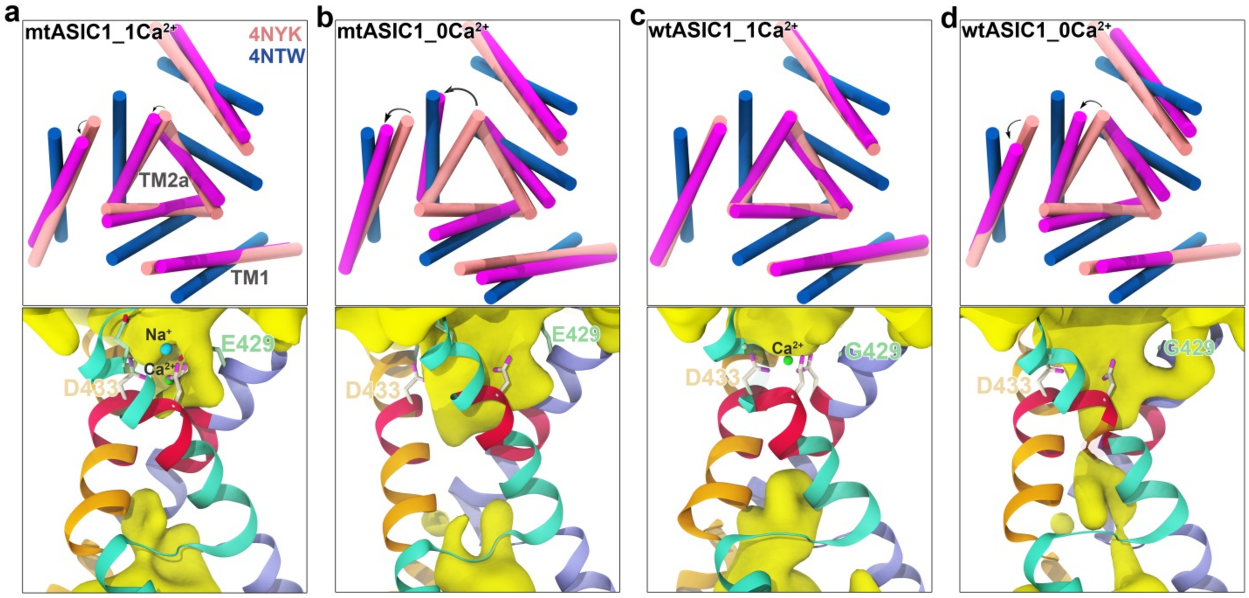

Supplementary Figure 2 | Extracellular view of the transmembrane (TM) domain showing the rotation of TM helices (upper panels) and side view of the TM domain illustrating the water density profile along the channel pore (lower panels). Panels **a** and **b** illustrate the results for the mutant simulations with or without Ca^{2+} bound; Panels **c** and **d** correspond to the wild type simulations with or without Ca^{2+} bound. In the upper panels, the pink and blue cylinders are with the two crystal structures in the desensitized and open states (PDB codes: 4NYK and 4NTW), respectively, while the magenta ones denote the representative conformations from the simulations. Note that for TM2, only the extracellular portion (TM2a) is displayed for clarity. The rotation direction and magnitude are indicated by a curved arrow. The water density maps shown in the lower panels are colored yellow. The Ca^{2+} and Na^{+} ions are depicted as green and cyan spheres, respectively; note that in the Ca^{2+}-free mutant system (panel **b**), the Na^{+} ions residing within the extracellular vestibule are masked by the contour map.

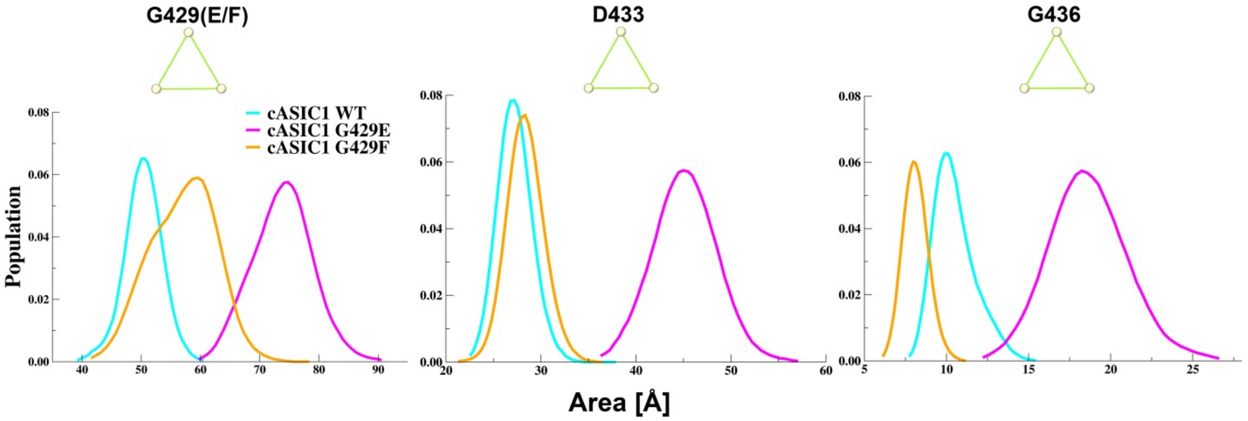

Supplementary Figure 3 | Overlap of the histograms for the area of the triangle formed by the Cα atoms of symmetry-related residues at position 429 (left), 433 (middle) and 436 (right) in the wild type (cyan line), G429E (magenta line) and G429F (orange line) channels. G429E mutation results in more prominent pore opening than G429F, suggesting the charge not the size of the side chain at the identified position plays a more significant role for channel opening.

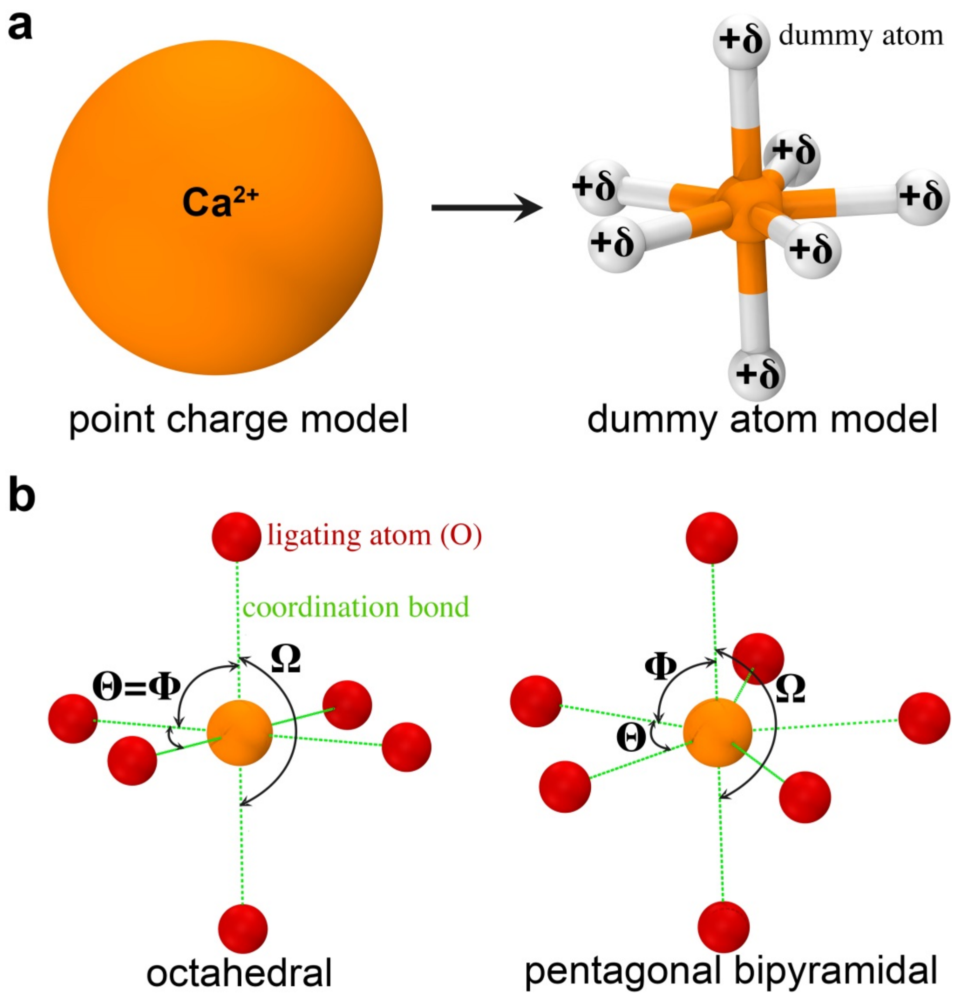

Supplementary Figure 4 | Schematic representation of the point charge and dummy atom models for Ca^{2+} used in the MD simulations (a) and illustration of the calculated angles for the octahedral and pentagonal bi-pyramidal coordination geometries (b). In cationic dummy atom model (or multi-site ion model) (**a**), the metal core is covalently connected to a certain number of cationic dummy atoms with a fractional charge of +δ (colored white) and the total charge is equivalent to the formal charge carried by the single-atom ion. For a perfect octahedral coordination, the angles **Θ/Φ** (12 in total) and **Ω (**3 in total) are equal to 90º and 180º, respectively, while the pentagonal bi-pyramidal geometry includes three distinct angles, **Θ** (5 in total), **Φ** (10 in total) and **Ω** (1 in total), with an ideal value of 72º, 90º and 180º, respectively.

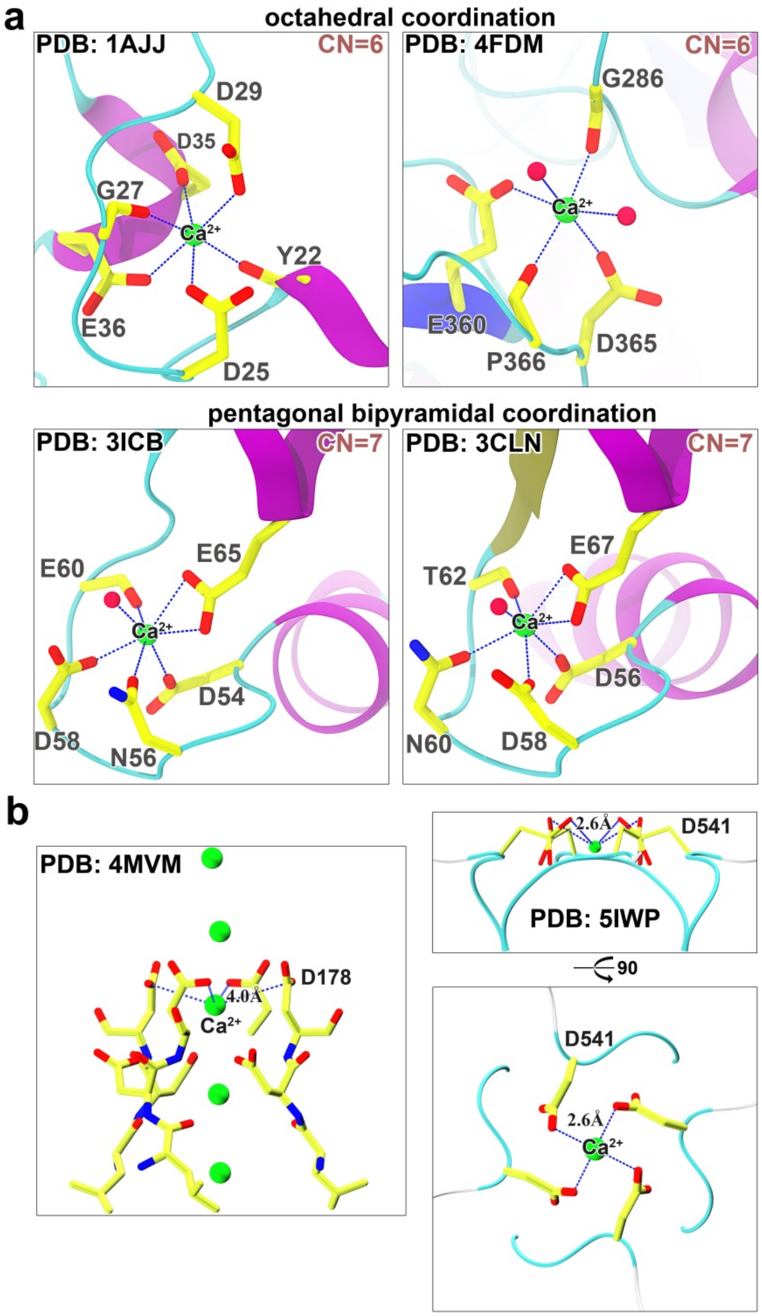

Supplementary Figure 5 | Ca^{2+} coordination geometries observed in selected soluble Ca^{2+} binding proteins (a) and ion channels (b). 1AJJ, low-density lipoprotein receptor ligand-binding module 5 solved at 1.7 Å; 4FDM, thermostable L2 lipase solvated at 1.6 Å; 3ICB, bovine intestinal calcium-binding protein solved at 2.3 Å; 3CLN, mammalian calmodulin solved at 2.2 Å; 4MVM, genetically engineered voltage-gated calcium channel Ca_vAb solvated at 3.2 Å; 5IWP, epithelial Ca^{2+}-selective transient receptor potential (TRP) channel TRPV6 solved at 3.65 Å. 3ICB and 3CLN contain characteristic EF hands. In the two C4 symmetric ion channels, the minimal distances between the trapped Ca^{2+} and the aspartate side-chain oxygens are indicated.

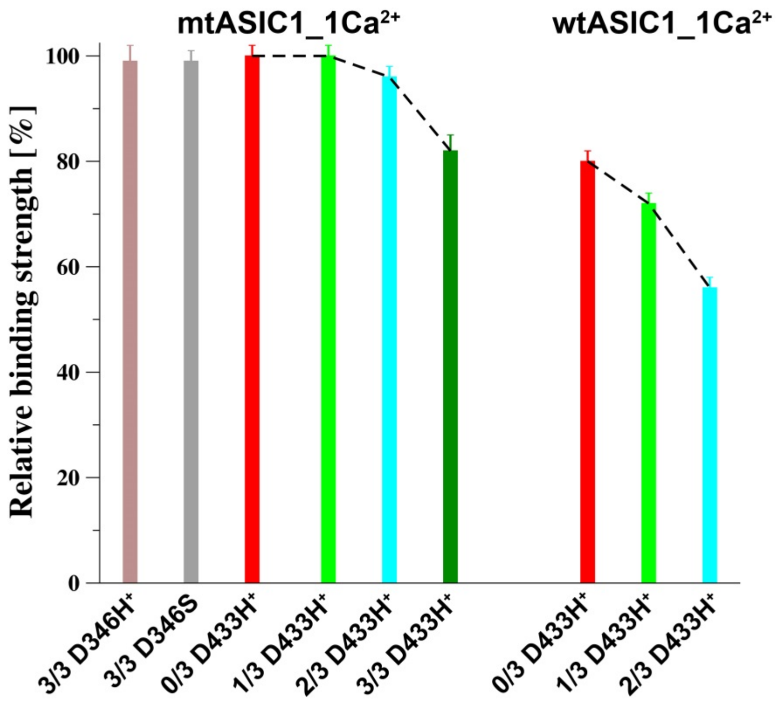

Supplementary Figure 6 | Comparison of the relative Ca^{2+} binding affinities for the different protonation states at the Asp433 cluster evaluated by the MM-GBSA approach. The results with the G429E mutant are presented on the left side, and those with the wild type on the right side. The data from the simulations with additional protonation at Asp346 or D346S mutation are also depicted (see the main text and **Supplementary Table 1**). The last 300 ns of the trajectory was taken for calculations (see **Methods**), involving 6,000 snapshots. Error bar denotes the standard deviation.

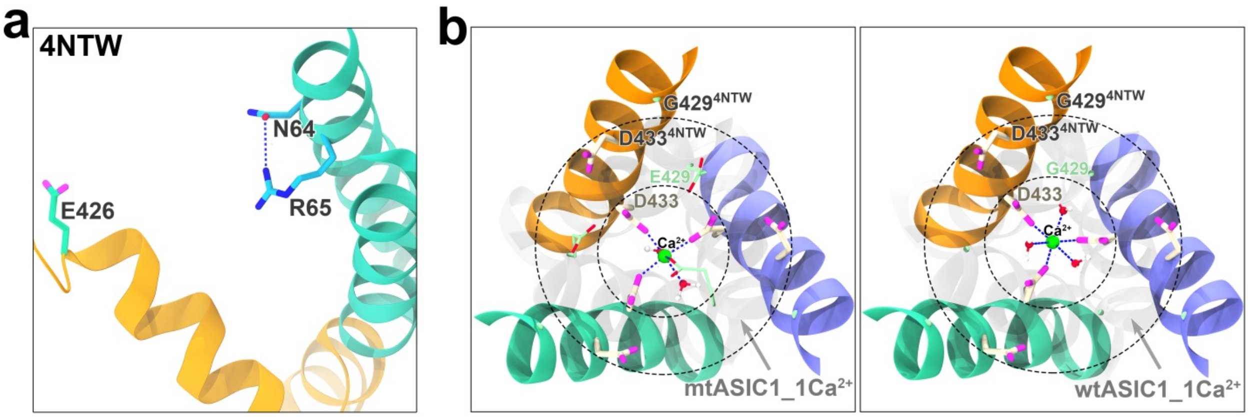

Supplementary Figure 7 | Disengagement of Glu426 and Arg65 in the MitTx-bound open state of cASIC1 (a) and extracellular view of the identified Ca^{2+} block sites in the cASIC1 G429E (left) and wild type (right) systems mapped onto the open state structure (b). In panel **b**, the channel pore is represented as transparent ribbons for the representative simulation snapshots. The residues Asp433 and Glu429 are shown as a stick model, and the Cα atom of Gly429 as a sphere. The inner circle approximately outlines the Ca^{2+} block site identified in the simulations using Asp433 as a landmark. It is apparent that the acidic residue ring in the open channel (outer circle) is out of reach of the docked Ca^{2+} for coordination without a conformational change.

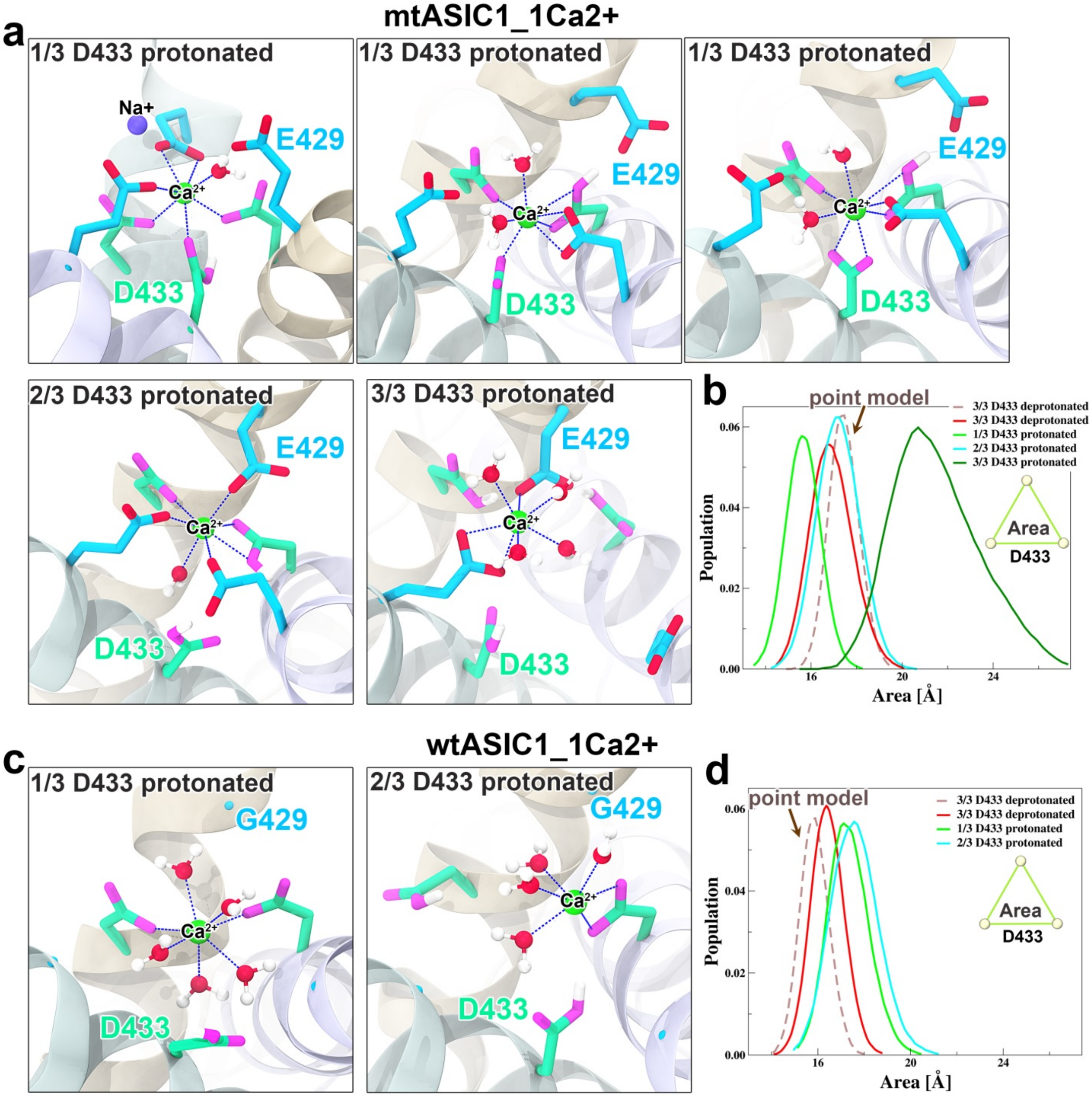

Supplementary Figure 8 | Comparison of the Ca^{2+} coordination patterns (a,c) and overlap of the histograms for the area formed by the gating residues Asp433 (b,d) derived with different protonation states at the Asp433 cluster in the mutant (upper) and wild type channels (lower). The coloring scheme and drawing style in panels **a** and **c** are the same to that of **Figs. 3c-3d**. The data were obtained from the simulations with the dummy atom model, except those explicitly indicated in panels **b** and **d** that were derived with the point charge model for comparison herein (see **Supplementary Table 1** and **Supplementary Text**). For clarity, the dummy complex has been represented as a Lennard-Jones sphere.

Supplementary Table 1 | Summary of the Molecular Dynamics Simulations Performed in This Study[§]

Set	Simulation System	Ca^{2+}[¶]	Protonation status[†]	Ca^{2+} model[‡]	Force fields	# of runs	Time/run [ns]	Data location
**S1**	cASIC1 G429E	1	3/3 Asp433 deprotonated	Point charge	CHARMM	2	1,000	Fig. 3b,c,e<br>Suppl. Fig. 2-3<br>Suppl. Fig. 6-7<br>Suppl. Fig. 8b,d
**S2**		0	3/3 Asp433 deprotonated	N.A.				
**S3**	cASIC1 Wild Type	1	3/3 Asp433 deprotonated	Point charge				
**S4**		0	3/3 Asp433 deprotonated	N.A.				
**S5**	cASIC1 G429E	1	3/3 Asp433 deprotonated	Dummy atom	AMBER	2	1,000	Fig. 3d<br>Suppl. Fig. 6<br>Suppl. Fig. 8
**S6**			1/3 Asp433 protonated					
**S7**			2/3 Asp433 protonated					
**S8**			3/3 Asp433 protonated					
**S9**	cASIC1 Wild Type	1	3/3 Asp433 deprotonated	Dummy atom		1		Fig. 3d<br>Suppl. Fig. 6<br>Suppl. Fig. 8
**S10**			1/3 Asp433 protonated					
**S11**			2/3 Asp433 protonated					
**S12**	cASIC1 G429F	0	3/3 Asp433 deprotonated	N.A.		2		Suppl. Fig. 3
**S13**	cASIC1 G429E	1	3/3 Asp433 deprotonated<br>3/3 Asp346 protonated	Dummy atom		2		Suppl. Fig. 3<br>Suppl. Fig. 6
**S14**	cASIC1 G429E/D346S	1	3/3 Asp433 deprotonated	Dummy atom				

§ S1 to S4 correspond to the simulations using CHARMM force field (plus Ca^{2+} dummy atom model, if applicable) (highlighted in light grey background). The remainders (viz., S5 to S14) were performed with AMBER force field (plus Ca^{2+} point charge model, if applicable). The charged form of Glu429 was used in all G429E mutant systems.

¶ "0" and "1" denote Ca^{2+}-free and Ca^{2+}-bound simulation systems, respectively.

† 1/3, 2/3 and 3/3 indicate single, double and triple protonation among the symmetry-related residues, respectively. The protonation states of the ionizable residues beyond the transmembrane domain were predicted by the program H++ as mentioned in **Methods**.

‡ The Ca^{2+} point charge model was developed by Marchand and Roux[4] (default in CHARMM),
and the Ca^{2+} catinonic dummy atom model was offered by Saxena and Sept[5].

Supplementary Table 2 | Ca^{2+} Coordination Geometries Calculated from the Molecular Dynamics Simulation Trajectories

Set[§]	Simulation System	CN[¶]	D(Ca^{2+}-OW)[†] [Å]	D(Ca^{2+}-OA)[†] [Å]	Θ(O-Ca^{2+}-O)[‡] [º]	Φ(O-Ca^{2+}-O)[‡] [º]	Ω(O-Ca^{2+}-O)[‡] [º]
**S1**	cASIC1 G429E	6	2.32 (0.1)	2.12 (0.1)	89.59 (10.9)		162.80 (7.2)
		7	2.40 (0.1)	2.20 (0.1)	72.62 (10.0)	90.00 (8.2)	171.50 (4.8)
**S3**	cASIC1 WT	6	2.28 (0.1)	2.11 (0.0)	89.66 (9.2)		164.20 (5.5)
**S5**	cASIC1 G429E	6	2.30 (0.1)	2.19 (0.0)	89.77 (9.1)		165.25 (6.0)
		7	2.36 (0.1)	2.23 (0.1)	72.40 (9.7)	89.63 (7.5)	165.96 (3.3)
**S6**		7	2.28 (0.0)	2.26 (0.1)	72.10 (9.3)	90.14 (7.0)	169.36 (4.9)
		8[*]	2.34 (0.1)	2.34 (0.2)	N.C.		
**S7**		7	2.26 (0.0)	2.28 (0.1)	72.49 (10.6)	89.30 (9.2)	162.62 (5.3)
**S8**		7	2.32 (0.1)	2.25 (0.1)	73.04 (7.3)	90.32 (10.0)	165.89 (7.3)
**S9**	cASIC1 Wild Type	6	2.29 (0.1)	2.17 (0.0)	89.80 (8.1)		166.70 (5.7)
**S10**		7	2.34 (0.1)	2.20 (0.1)	73.18 (3.3)	90.04 (11.0)	166.04 (4.7)
**S11**		6[#]	2.32 (0.1)	2.29 (0.1)	72.43 (9.5)	88.38 (9.2)	N.A.
**S13**	cASIC1 G429E	6	2.33 (0.1)	2.19 (0.0)	89.80 (9.0)		165.08 (5.8)
**S14**	cASIC1 G429E/D346S	7	2.33 (0.1)	2.24 (0.1)	73.55 (10.5)	91.22 (9.3)	164.57 (3.9)

§ See **Supplementary Table 1** for more details regarding each simulation system

¶ Coordination number [determined with a distance cutoff of 2.6 Å for CN<8 or 2.8 Å for CN=8]

† Coordination bond length. OW, water molecule oxygen; OA, amino acid oxygen. The experimental Ca^{2+}-OW distance is 2.35~2.46 Å, depending on the coordination number[6-8].

‡ Coordination angle. See **Supplementary Fig. 4** for illustration of each angle.

* Distorted square anti-prismatic (SAP) geometry; N.C., angles not calculated.

\# Pentagonal (mono)pyramidal geometry; N.A., angle not available.

Supplementary Table 3 | Site-Directed Mutagenesis Primers

Receptor	Primer
cASIC1 G429E forward	5' – GGCGTATGAGGTGGCTGAGTTGCTGGGTGACATCG – 3'
cASIC1 G429E reverse	5' – CGATGTCACCCAGCAACTCAGCCACCTCATACGCC – 3'
rASIC3 E435G forward	5' – GGCCTATGAAGTGTCGGGACTGCTGGGAGACATTG – 3'
rASIC3 E435G reverse	5' – CAATGTCTCCCAGCAGTCCCGACACTTCATAGGCC – 3'

Supplementary Note 1: Comparison of the two Ca^{2+} models

In this study, two distinct Ca^{2+} models[4,9] were used in the simulations of Ca^{2+}-bound channel systems within the context of the additive CHARMM or AMBER force fields (**Methods**). One is the most common point charge model, which describes the ion as a formal point charge of +2 that interacts with the ligands through non-bonded interactions. The other one is the more complicated cationic dummy atom model (originally developed by Åqvist and Warshel[9,10]), which represents partially covalent and partially electrostatic nature of the coordinative bond by locally splitting up the space between the metal ion and the ligand into a covalent bond (between the metal core and the cationic dummy atom) and an electrostatic interaction (between the cationic dummy atom and the partially negatively charged ligand). Despite the different development strategies, our simulation results showed that the two Ca^{2+} models produced high structural homogeneity at the channel binding site. In the case of the mutant system, the two differently represented ions exploited the same numbers of the carboxylate oxygens and the water oxygens for an optimal coordination (**Figs. 3c-3d**; **Supplementary Table 2**), though the specific spatial arrangements of these ligating oxygens show a difference. In the wild system, the two models achieved essentially the same coordination patterns inside the channel pore (**Figs. 3c-3d**; **Supplementary Table 2**). Moreover, comparable pore openings were observed with the two models in both the mutant and wild type channels (**Supplementary Figs. 8b and 8d**).

Yet, our MM-GBSA calculations displayed a difference of ~160 kcal/mol between the Ca^{2+} binding free energies estimated from the two models for either the mutant and wild type systems. The discrepancy is mainly caused by the distinct electrostatic description used in the two models. It should be noted that, herein we are most interested in the overall trend of the Ca^{2+} binding affinity changes as a result of varied protonation state within the same system as well as in the relative change in binding affinity due to mutation, rather than seeking the ideal absolute values. With the dummy atom model, the simulation results demonstrated an overall progressive reduction in the Ca^{2+} binding

strength with increasing protons at the block site, supporting the previous argument that Ca^{2+} and proton compete for the binding site[11]. In addition, both the Ca^{2+} models showed an enhanced Ca^{2+} binding affinity through the G429E mutation, as we expected. The dummy model gave an increase of ~20% (**Supplementary Fig. 6**), as compared to that of ~40% with the point charge model (data not depicted). Again, we reason that this difference is related to the different electrostatic treatments between the two models. Yet, it does not qualitatively influence our conclusions.

The Ca^{2+} dummy model used herein was developed against the most common pentagonal bi-pyramidal geometry in biological systems[5], involving a coordination number (CN) of 7 (**Supplementary Fig. 4a**). In our simulations, interestingly, we discovered that this model is also capable of forming the 6-lignad or 8-ligand coordination (**Fig. 3c**; **Supplementary Fig. 8a**). The observations thus highlight the geometric flexibility of the Ca^{2+} dummy model and its ability in simulating the process involving CN changes, as also indicated in other studies[12,13].

Supplementary References

1 Larkin, M. A. *et al.* Clustal W and clustal X version 2.0. *Bioinformatics* **23**, 2947-2948 (2007).

2 Robert, X. & Gouet, P. Deciphering key features in protein structures with the new ENDscript server. *Nucleic Acids Res.* **42**, W320-W324 (2014).

3 Gonzales, E. B., Kawate, T. & Gouaux, E. Pore architecture and ion sites in acid-sensing ion channels and P2X receptors. *Nature* **460**, 599-U562 (2009).

4 Marchand, S. & Roux, B. Molecular dynamics study of calbindin D9k in the apo and singly and doubly calcium-loaded states. *Proteins Structure Function and Genetics* **33**, 265-284 (1998).

5 Saxena, A. & Sept, D. Multisite ion models that improve coordination and free energy calculations in molecular dynamics simulations. *J. Chem. Theory Comput.* **9**, 3538-3542 (2013).

6 Probst, M., Radnai, T., Heinzinger, K., Bopp, P. & Rode, B. Molecular dynamics and x-ray investigation of an aqueous calcium chloride solution. *The Journal of Physical Chemistry* **89**, 753-759 (1985).

7 Jalilehvand, F. *et al.* Hydration of the calcium ion. An EXAFS, large-angle X-ray scattering, and molecular dynamics simulation study. *J. Am. Chem. Soc.* **123**, 431-441 (2001).

8 Katz, A. K., Glusker, J. P., Beebe, S. A. & Bock, C. W. Calcium ion coordination: a comparison with that of beryllium, magnesium, and zinc. *J. Am. Chem. Soc.* **118**, 5752-5763 (1996).

9 Oelschlaeger, P., Klahn, M., Beard, W. A., Wilson, S. H. & Warshel, A. Magnesium-cationic dummy atom molecules enhance representation of DNA polymerase β in molecular dynamics simulations: Improved accuracy in studies of structural features and mutational effects. *J. Mol. Biol.* **366**, 687-701 (2007).

10 Aaqvist, J. & Warshel, A. Free energy relationships in metalloenzyme-catalyzed reactions. Calculations of the effects of metal ion substitutions in staphylococcal nuclease. *J. Am. Chem. Soc.* **112**, 2860-2868 (1990).

11 Immke, D. C. & McCleskey, E. W. Protons open acid-sensing ion channels by catalyzing relief of Ca2+ blockade. *Neuron* **37**, 75-84 (2003).

12 Duarte, F. *et al.* Force field independent metal parameters using a nonbonded dummy model. *The Journal of Physical Chemistry B* **118**, 4351-4362 (2014).

13 Åqvist, J. & Warshel, A. Computer simulation of the initial proton transfer step in human carbonic anhydrase I. *J. Mol. Biol.* **224**, 7-14 (1992).
